# Mycosporine-Like Amino Acids from Marine Resource

**DOI:** 10.3390/md19010018

**Published:** 2021-01-04

**Authors:** Félix L. Figueroa

**Affiliations:** Institute for Blue Biotechnology and Development (IBYDA), Malaga University, Campus Universitario de Teatinos s/n, E-29071 Malaga, Spain; felix_lopez@uma.es; Tel.: +34-9-5213-1672

**Keywords:** antioxidant, chemical identification, data base of MAAs, extraction, macroalgae, HPLC, mass spectroscopy, mycosporine-like amino acids, zooplankton

## Abstract

In the last 10 years, a great number of publications (both regular papers and reviews) have been published on the interesting molecules—mycosporine-like amino acids (MAAs). Despite significant advances in the research of MAAs, current overviews in the recent publications involving MAA research still need reporting. The aim of this Special Issue is to join, as an interdisciplinary approach, the photochemical and photobiological aspects, with emphasis on new natural resources to obtain both algae and zooplankton MAAs, advances in methodology of extraction and chemical identification of new MAAs. Finally, this Special Issue reviews the bioactivities of MAAs including UVR screen, antioxidant, immunostimulant, growth factor, DNA protection, inhibition of collagenase, elastase and hyaluronidase, and anti-photoaging, among others, and their potential use as nutracosmeceutic molecules (i.e., oral and topic photoprotector).

## 1. Introduction

Mycosporine-like amino acids (MAAs) are low molecular weight molecules that are water-soluble, nitrogen enriched and have absorption maxima in the UV region (310–365 nm). They are ideal sunscreens due to their high photo- and thermostability, strong UV absorption, energy dissipation as heat and short-lived excited state avoiding unwanted photochemical reactions as photoproduct formation. They have been detected in cyanobacteria, microalgae, macroalgae (mainly in Rhodophyta), and marine animals (by ingestion). Their UV- absorption, antioxidant capacity and physico-chemical features give MAAs a potential to be used in applications for the prevention and therapeutic treatment of illnesses related to free-radical production and UV irradiation in humans.

A great number of regular papers, reviews and books have been published on MAAs indicating the interest not only at a basic research level but also in the transfer of new advances to the cosmeceutical industry [1,2,3,4,5,6,7,8,9,10,11] Sunscreens based on MAAs are available on the market, using porphyra-334 and shinorine, but these compounds have been isolated from one unique species of genus *Porphyra*. Schmid et al. [12] developed a cream containing the liposomal porphyra-334 and shinorine has been commercialized as Helioguard^®^365. They found that apart from a high anti-aging activity, the formulation exhibits protective properties against UV-A-induced loss of cell viability and DNA damage. Helioguard^®^365 exhibits a high preventive effectiveness against UV-A-caused damage to the human skin, i.e., the skin firmness and skin smoothness were improved after the application of Helioguard^®^365, as compared to untreated areas of the skin or a cream control [13]. Helionori^®^ is other product offering natural protection against sunburning, containing MAAs as active ingredients, namely, porphyra-334 and shinorine—extracted from the *P. umbilicalis*. Helionori^®^ (2%) strongly preserved membrane lipids of keratinocytes by 139% and fibroblasts by 134%, as well as offered maximal protection for DNA [14]. More recently, another MAA, Palythine, extracted from the red alga *Chondrus yendoi*, has been shown to have high photoprotective capacity in HaCaT human keratinocytes after testing cell viability, DNA damage (nonspecific, cyclobutane pyrimidine dimers and oxidatively generated damage) and gene expression changes (linked to inflammation, photoageing and oxidative stress) and antioxidant activity [15]. Palythine offered statistically significant protection (*p* < 0.005) against all end points tested even at extremely low concentrations (0.3% *w/v*) and in addition it presents potent antioxidant capacity [15]. Thus, porphyra-334, shinorine and palythine present effective multifunctional photoprotective properties in vitro and have the potential to be developed as a natural and biocompatible alternative to currently approved UVR filters. This is an important point since The European Chemicals Agency (ECHA) is concerned about the potential adverse health and ecotoxical effects of eight of sixteen commonly used sunscreen filters in Europe. The Environmental Effects Assessment Panel (EEAP) of the United Nations Environment Program has expressed similar concerns. The safety of UV filters for sunscreens is determined by toxicological studies, such as acute oral toxicity, chronic toxicity, embryofetal toxicity dermal toxicity, photo-irritation, and percutaneous absorption [16]. Many efforts have been made to develop sunscreens with a broad absorption spectrum and no toxicity, which allows them to absorb both UV-A and UV-B radiation, without the need for high amounts of chemicals, because some have been associated with allergic reactions or phototoxicity [17]. Certain UV filters can affect human health as endocrine disruption properties [18], skin penetration [19], low photostability, low biodegradability and lack of effectiveness in skin protection [20]. The present commercial inorganic and organic particulate UV filters can provoke damage in the natural environment [21,22]. Chemical sunscreens are accumulating in coastal and continental waters [23] and they can cause a rapid complete bleaching of hard corals, even at extremely low concentrations [24]. UV filters have been found in invertebrates and fishes [21,25,26]. In addition, Sánchez-Quiles and Tóvar-Sánchez [22] showed that inorganic oxide nanoparticles with the UV filter TiO_2_, produce hydrogen peroxide in coastal waters, concluding that TiO_2_ nanoparticles are the major oxidizing agent entering coastal waters in touristic areas with direct ecological consequences on the ecosystems.

Thus, it is important to develop new materials as UV filters with higher photostability and biodegradability and no toxic effects, both for humans and the whole ecosystem. Among these candidates, MAAs are an alternative to chemical synthetic substances since they are filters obtained from natural resources without any reported toxicity and they have high photostability and thermostability [12,27]. However, they have not yet been broadly exploited at a commercial scale and only a few products are available, such as Helioguard^®^365 and Helionori^®^ which include MAAs extracted from *Porphyra umbilicalis.* In the future, advances in the development of new cosmeceutical products containing MAAs obtained from other marine resources, other than *P. umbilicalis* are expected.

This Special Issue “Mycosporine-Like Amino Acids from Marine Resource” presents several chapters on advances in methodology for extraction and chemical identification of MAAs from different algae. It is necessary to investigate new natural resources containing high contents of MAAs and specific compositions of MAAs among the pool of known or new molecules with the highest antioxidant capacity [8,28,29,30]. In this Special Issue, several papers on the distribution of MAAs among marine organisms such as macroalgae and zooplankton are presented. Finally, MAAs as sunscreens due to their UV photoprotection, antioxidant and anti-photoaging properties are reviewed in other manuscripts. This Special Issue intends to contribute to the advancement of the research on MAAs, adding information on these potent photoprotective substances due to their UV-screen, antioxidative, DNA protection, anti-inflammatory and anti-aging properties [9,11]

## 2. Methodology for Extraction and Chemical Identification of MAAs

There are several reported protocols for extraction using different solvents, temperatures and extraction times. Karsten et al. [31] evaluated the effect of re-dissolution solvents (100% methanol, distilled water, and HPLC eluent), after dryness, on the MAA extraction efficiency using different HPLC columns (Synergi C18, Sphereclone C8, and Luna C8). Distilled water and the HPLC eluent gave almost identical peak patterns and MAA contents on the C8 and C18 columns [31]. In contrast, the application of the widely used methanol, led to double peaks or even the loss of specifc peaks, as well as to a strong decline in total MAA amounts ranging from about 35% of the maximum in *P. crispa* to 80% of the maximum in *P. umbilicalis* [31]. Consequently, Karsten et al. [31] suggested that methanol should be avoided as a re-dissolution solvent for the HPLC sample preparation. The protocol for extraction and HPLC identification based on the C18 column by Karsten et al. [31] is compared with protocols reported in three papers in this issue [32,33,34].

Chaves-Peña et al. [32], in this issue, compared MAA extraction using distilled water and 20% aqueous methanol in four Rhodophyta. Different re-dissolution solvents and C8 and C18 columns were tested for the HPLC analysis. Porphyra-334, shinorine, palythine, palythine-serine, asterina-330, and palythinol were identified by HPLC/ESI-MS. The separation of these MAAs was improved by employing the C8-column, and using methanol as a re-dissolution solvent. Regarding the total MAA concentrations, no differences between the two solvents were found but the highest MAA amounts were observed by injecting them directly in the HPLC. According to these results, distilled water could be an excellent extraction solvent for MAAs, as Nishida et al. [33] concluded in the extraction of MAAs form *Palmaria palmata*. Nishida et al. [33] applied a successive extraction method by using water and then methanol extraction, and spectrophotometric and HPLC analyses revealed that the yield of MAAs by 6 h water extraction was the highest among the tested conditions. Nevertheless, according to Chaves-Peña et al. [32] the re-dissolution in pure methanol after dryness was the best option for the qualitative analysis of the most common MAAs in red algae in contrast to those reported by Karsten et al. [31]. The efficient extraction in water has advantages for the use of MAAs in natural cosmetics since methanol is a reactive that is not allowed in natural cosmetics.

On the other hand, Orfanoudaki et al. [34] identified seven mycosporine like-amino acids and two betaines were isolated from salt marsh collected red alga *Bostrychia scorpioides* using various chromatographic techniques. Their structures were confirmed by nuclear magnetic resonance (NMR) spectroscopy and high resolution mass spectrometry (HRMS). Six MAAs and one betaine were chemically characterized as new natural products. The identification of new MAAs open the opportunity for research on their bioactivity, especially to evaluate their antioxidant and anti-inflammatory properties. Orfanoudaki et al. [34] presented the absolute configuration of 14 mycosporine-like-amino acids extracted from *Bostrychia scorpiodes*, determined by combining the results of electronic circular dichroism (ECD) experiments and those of advanced Marfey’s method using LC-MS. The crystal structure of a shinorine hydrate was determined from a single crystal X-ray diffraction study and its absolute configuration was established from anomalous-dispersion effects.

## 3. Distribution of MAAs among Marine Organisms: Macroalgae and Zooplankton

Many studies assessing the MAA concentration and composition have been realized in species from different environments around the world—from tropical to polar region. This screening is an effort aimed at finding species with high MAA concentrations and a high and sustainable year-round production of biomass. In order to find new natural molecules with photoprotective properties, it is very important to conduct screening from natural resources as has been conducted in the last years [34,35,36,37,38,39,40,41]. In the screening studies, it is possible to identify the species with the highest content of MAAs. The MAA contents in the algae growing in coastal waters are affected mainly by irradiance and nitrate levels and thus the MAA level is affected by the season [40,41].

In the Chilean coast (temperate region), the highest MAA concentrations were reached in species of the genus *Porphyra* (2 to 10 mg g^−1^ DW), following by *Bostrychia* (4.7 mg g^−1^ DW) [35]. Hoyer et al. [36] reported that from 17 red algae species studied the endemic to Antarctica species *Porphyra endiviifolium* (9.7 mg g^−1^ DW), *Bangia atropurpurea* (5.8 mg g^−1^ DW) and *Curdiea racowitzae* (4.9 mg g^−1^ DW) showed the highest MAA concentration. In the European coast, the highest concentration of MAAs were detected in *Gymnogongrus devoniensis* (1.5–7.8 mg g^−1^ DW), followed by *Ceramium nodulosum* (7.6 mg g^−1^ DW), *Bangia atropurpurea* (5.5–7 mg g^−1^ DW) and *Gelidium pusillum* (5–6.5 mg g^−1^ DW) [37,38]. Karsten et al. [39] studied the MAA concentration from 18 red algae species, reporting the highest MAA concentration in *Bostrychia radicans* (2.9–12 mg g^−1^ DW), *Stictosiphonia arbuscula* (6 mg g^−1^ dw)*, Caloglossa leprieurii* (2–6.5 mg·g^−1^ DW) and *Catenella impudica* (5.2 mg g^−1^ DW). In the coastal waters of Brazil, the highest content of MAAs was found in *Pyropia acantohora* (5.9 mg g^−1^ DW) followed by *Hypnea musciformis* (3 mg g^−1^ DW) and *Spyridia clavata* (2 mg g^−1^ DW) [40]. The highest content was not reached in areas with the highest UVR dose (tropical areas), but in coastal waters of subtropical nitrate enriched areas due to coastal upwelling [40]. Schneider et al. [41) reported the highest levels of MAAs in algae collected from Mediterranean and Atlantic coasts of the southern Iberian Peninsula in *Porphyra umbilicalis* (11 mg g^−1^ DW), *Bangia atroporpurea* (5.5 mg g^−1^ DW), *Felmanophycus rayssiae* and *Porphyra leucosticta* (4 mg g^−1^ DW) Thus, the highest content of MAAs is found in species of the Bangiales order of the genus *Pophyra, Pyropia* or *Bangia.*

Sun et al. [42], in this issue, presented a database of MAAs of macroalgae (http://210.28.32.218/MAAs/) based on CiteSpace software used on the Web of Science, Springer, Google Scholar, and China national knowledge infrastructure (CNKI). Previously, Sinha et al. [43] presented a database of mycosporines and MAAs in fungi, cyanobacteria, phytoplankton, macroalgae and animals. The study by Sun et al. [42] summarized and analyzed the papers related to MAAs in marine macroalgae over the past 30 years (1990–2019), mainly focused on MAA distribution, contents, and types. It was confirmed that 572 species marine macroalgae contained MAAs, namely in 45 species of Chlorophytes, 41 species of Phaeophytes, and 486 species of Rhodophytes, and they, respectively, belonged to 28 orders. An open online database to quickly retrieve MAAs in 501 species of marine macroalgae is presented. In any case, the identification has been reported following different techniques such as HPLS, ESI-mass spectroscopy and RNM. By using only HPLC, is not possible to obtain a precise identification, thus it is necessary in the chemical identification studies to include data of ESI-mass spectroscopy or RNM. On the other hand, MAA standards by purification of MAAs from natural resource to be used in the chemical identification, are not still available on the market. Thus, it is necessary to strengthen the research in the preparation and purification of MAA purified standards from marine macroalgae in the future in order to advance in the quantification of different MAAs from natural resources.

Among the organisms with MAAs, Hylander [44] shows in this Special Issue that zooplankton MAA concentrations range from non-detectable to ~13 mg g DW^−1^. The last, is close to the highest level found in macroalgae (order Bangiales). Copepods, rotifers, and krill display a large range of concentrations, whereas cladocerans generally do not contain MAAs. The proposed mechanisms to gain MAAs are via ingestion of MAA-rich food or via symbiotic bacteria providing zooplankton with MAAs. Exposure to UV-radiation increases the concentrations in zooplankton, both via increasing MAA concentrations in phytoplankton food and due to active accumulation. MAA content in zooplankton is affected by the season, being generally low during winter and higher in summer. Females seem to deposit MAAs in their eggs. In addition, MAAs in zooplankton increase with altitude but only up to a certain altitude suggesting some limitation for the uptake. A high MAA concentration has also been shown to lead to lower UV-induced mortality and an overall increased fitness.

In this issue, Jofre et al. [45] shows that the content and proportion of the composition of MAAs vary depending on the species and several environmental factors. Its high cosmetic interest calls for research on the content and composition. By using spectrophotometric and HPLC techniques, the content and composition of MAAs of intertidal sub-Antarctic red macroalgae *Iridaea tuberculosa*, *Nothogenia fastigiate*, and *Corallina officinalis* were assessed. Both content and composition of MAAs varied seasonally. *I. tuberculosa* exhibited the highest MAA values (above 1 mg g^−1^ of dried mass weight), porphyra-334 was the main component in *N. fastigiata*, whereas *I. tuberculosa* and *C. officinalis* exhibited a high content of palythine. Interestingly, these two MAAs, porphyra-334 and palythine, present high antioxidant activity [8,15,29]. Some samples were also analyzed using high-resolution mass spectrometry coupled with HPLC-ESI-MS in order to identify more precisely the MAA composition. HPLC-ESI-MS allowed us to identify seven different MAAs. Two were recorded for the first time in seaweeds from sub-Antarctic areas (mycosporine-glutamic acid and palythine-serine), and an eighth UV-absorbing compound which remains unidentified was also recorded [45].

Finally, Vega et al. [46] presents an screening among red amacroalgae and cyanobacteria of mycosporine like amino acids and other UV screen substances as Polyphenols and scytonemin (only presented in Cyanobacteria). The highest concentrations of MAAs were found in the red macroalgae *Porphyra umbilicalis*, *Gelidium corneum* and *Osmundea pinnatifida* and in the cyanobacterium *Lyngbya* sp. *Scytonema* sp. was the unique species that presented an MAA with maximum absorption in the UV-B band, being identified as mycosporine-glutaminol for the first time in this species [46]. Water was the best extraction solvent for MAAs and phenols, whereas scytonemin was better extracted in a less polar solvent such as ethanol:_d_H_2_O (4:1) and positive correlations of antioxidant activity with different molecules, especially polyphenols, biliproteins and MAAs, were observed [46]. Hydroethanolic extracts of some species incorporated in creams showed an increase in the photoprotection capacity in comparison with the base cream. Thus extracts of red macroalgae and cyanobacteria can be used as natural photoprotectors improving the diversity of sunscreens. The combination of different extracts enriched in scytonemin and MAAs could be useful to design broad-band natural UV-screen cosmeceutical products [46].

### MAAs as Sunscreens: Antioxidant and Anti-Photoaging Properties

In the last part of the Special Issue, Nishida et al. [33] analyzed MAAs in a seasonal study and found that both the highest antioxidant capacity, determined by ABTS methods, and the content of MAAs was reached in February (6.93 μmol g^−1^ DW). The highest scavenging activity and reducing power were found at alkaline condition (pH 8.0).

Orfanoudaki et al. [30] showed that the MAAs extracted from the red alga *Bostrychia scorpiodes* presented anti-aging and wound-healing properties by conducting three different assays, namely the inhibition of collagenase, inhibition of advanced glycation end products (AGEs) and wound healing assay (scratch assay).

Finally, Rosic [47] presented a review on MAAs as molecules to be used for skin protection. By scavenging ROS, MAAs play an antioxidant role and suppress singlet oxygen-induced damage. According to Rosic [47], currently, there are over 30 different MAAs found in nature and they are characterized by different antioxidative and UV-absorbing capacities. Depending on the environmental conditions and UV level, up- or down-regulation of genes from the MAA biosynthetic pathway results in seasonal fluctuation of the MAA content in aquatic species. The review by Rosic [46] provides a summary of the MAA antioxidative and UV-absorbing features, including the genes involved in the MAA biosynthesis. Specifically, regulatory mechanisms involved in MAAs pathways are evaluated for controlled MAA synthesis, advancing the potential use of MAAs in human skin protection. The active research on mycosporine-like amino acids will bring more findings on the usefulness in UVR photoprotection as sunscreens, activators of cells proliferation, anti-cancer agents, anti-photoaging molecules, stimulators of skin renewal and functional ingredients of UV-protective biomaterials [48].

## References

[1] Torres A., Enk C.D., Hochberg M., Srebnik M. (2006). Porphyra-334, a potential natural source for UVA protective sunscreens. Photochem. Photobiol. Sci..

[2] Kim S.K., Ravichandran Y.D., Khan S.B., Kim Y.T. (2008). Prospective of the cosmeceuticals derived from marine organisms. Biotechnol. Bioproc. Eng..

[3] Carreto J.I., Carrignan M.O. (2011). Mycosporine-like amino acids: Relevant Secondary Metabolites. Chemical and Ecological Aspects. Mar. Drugs.

[4] Bhatia S., Garf A., Sharma K., Kumar S., Sharma A., Purohit A.P. (2011). Mycosporine and mycosporine-like amino acids: A paramount tool against ultraviolet radiation. Pharm. Rev..

[5] Suh S.S., Hwang J., Park M., Seo H.H., Kim H.S., Lee J.H., Moh S.H., Lee T.K. (2014). Anti- inflammation of mycosporine like amino acid (MAAs) in response to UV radiation suggest potential anti-skin aging activity. Mar. Drugs.

[6] Hartmann A., Gostner J., Fuchs J.E., Chalta E., Aligiannis N., Skaltsounis L., Ganzera M. (2015). Inhibition of collagenase by mycosporine-like amino acids form marine sources. Planta Med..

[7] Lawrence K.P., Long P.F., Young A.R. (2017). Mycosporine-like Amino Acids for Skin Photoprotection. Curr. Med. Chem..

[8] Wada N., Sakamoto T., Matsugo S. (2015). Mycosporine-like Amino Acids and Their Derivatives as Natural Antioxidants. Antioxidants.

[9] Navarro N.P., Figueroa F.L., Korbee N., Bonomi J., Álvarez Gómez F., de la Coba F., Rastogi R.P. (2018). Mycosporine-like amino acids form red algae to develop natural UV sunscreens. Sunscreens: Source, Formulation, Efficacy and Recommendations.

[10] Orfanoudaki M., Hartmann A., Karsten U., Ganzera M. (2019). Chemical profiling of mycosporine-like amino acids in twenty-three red algal species. J. Phycol..

[11] Kageyama H., Waditee-Sirisattha R. (2019). Antioxidative, anti-inflammatory and anti-aging properties of mycosporine-like amino acids and cellular mechanisms in the protection of skin-aging. Mar. Drugs.

[12] Schmid D., Schürch C., Zülli F. (2006). Mycosporine-like amino acids from red algae protect against premature skin-aging. Euro Cosmet..

[13] Schmid D., Schürch C., Zülli F., Nissen H.P., Prieur H. (2003). Mycosporine-like amino acids: Natural UV-screening compounds from red algae to protect the skin against photoaging. SÖFW J..

[14] Andre G., Pellegrini M., Pellegrini L. (2001). Algal Extracts Containing Amino Acid Analogs of Mycosporine are Useful as Dermatological Protecting Agents against Ultraviolet Radiation.

[15] Lawerenze K.P., Gacesa R., Long P.F., Young A.R. (2017). Molecular photoprotection of human keratinocytes by the naturally ocurring mycosporine-like amino acid palythine. Br. J. Dermatol..

[16] Lim H.W., Draelos Z.D. (2009). Clinical Guide to Sunscreen and Photoprotection.

[17] Kawakami C.M., Gaspar L.R. (2015). Mangiferin and naringenin affect the photostability and phototoxicity of suncreens conating avobenzone. J. Photochem. Photobiol..

[18] Krause M., Klit A., Bomberg-Jensen M., Soeborg T., Frederiksen H., Schlumpf M., Lichtensteiger W., Skakkebaek N.E., Drzewiecki K.T. (2012). Sunscreens: Are they beneficial for health? An overview of endocrine disrupting properties of UV-filters. Int. J. Androl..

[19] Sarveiya V., Risk S., Benson H.A.E. (2004). Liquid chromatographic assay for common sunscreen agents: Application to in vivo assessment of skin penetration and systemic absorption in human volunteers. J. Chromatogr. B.

[20] Kockler J., Oelgemoller M., Robertson S., Glass D. (2012). Photostability of sunscreens. J. Photochem. Photobiol. C.

[21] Kaiser D., Sieratowicz A., Zielke H., Oetken M., Hollert H., Oehlmann J. (2012). Ecotoxicological effect characterization of widely used organic UV filters. Environ. Pollut..

[22] Sánchez-Quiles D., Tovar-Sánchez A. (2014). Sunscreens as a source of Hydrogen Peroxide production in coastal waters. Environ. Sci. Technol..

[23] Fent K., Zenker A., Rapp M. (2010). Widespread occurrence of estrogenic UV filters in aquatic ecosystems in Switzerland. Environ. Pollut..

[24] Danovaro R., Bongiorni L., Corinaldesi C., Giovannelli D., Damiani E., Astolfi P., Greci L., Pusceddu A. (2008). Sunscreens cause coral bleaching by promoting viral infections. Environ. Health Perspect..

[25] Balmer M.E., Buser H.R., Muller M.D., Poiger P. (2005). Occurrence of some organic UV filters in wastewater, in surface waters, and in fish from Swiss lakes. Environ. Sci. Technol..

[26] Kuntz P.Y., Gries T., Fent K. (2006). The ultraviolet filter 3-Benylidine camphor adversely affects reproduction in fathead minnow (*Pimephales promelas*). Toxicol. Sci..

[27] Fernandes S.C.M., Alonso-Varona A., Palomares T., Zubillaga V., Labidi J., Bulone V. (2015). Exploiting Mycosporines as natural sunscreens for the fabrication of UV-absorbing Green materials. Appl. Mater. Int..

[28] De la Coba F., Aguilera J., de Gálvez M.V., Álvarez M., Gallego E., Figueroa F.L., Herrera E. (2009). Prevention of the ultraviolet effect in clinical and histopathological changes, as well as the heat shock protein 72 expression in mouse skin by topical application of algal UV absorbing compounds. J. Dermatol. Sci..

[29] De la Coba F., Aguilera J., Figueroa F.L., de Gálvez M.V., Herrera E. (2009). Antioxidant activity of mycosporine-like amino acids isolated form three red macroalgae and one marine lichen. J. Appl. Phycol..

[30] Orfanoudaki M., Hartmann A., Aliou M., Gelbrich T., Blanchard P., Derbré S., Schinkovitz A., Richomme P., Hensel A., Ganzera M. (2020). Absolute configuration of mycosporine-like amino acids, their wound healing properties and in vitro anti-ageing effects. Mar. Drugs.

[31] Karsten U., Escoubeyrou K., Charles F. (2009). The effect of re-dissolution solvents and HPLC columns on the analysis of mycosporine-like amino acids in the eulittoral macroalgae *Prasiola crispa* and *Porphyra umbilicalis*. Helgol. Mar. Res..

[32] Chaves-Peña P., de la Coba F., Figueroa F.L., Korbee N. (2020). Quantitative and qualitative HPLC analysis of Mycosporine-like amino acids extracted in distilled water for cosmetical uses in four Rhodophyta. Mar. Drugs.

[33] Nishida Y., Kumagi Y., Michiba S., Yasui H., Kishimura H. (2020). Efficient extraction and antioxidant capacity of mycosporine-like amino acids from red alga Dulse *Palmaria palmata* in Japan. Mar. Drugs.

[34] Orfanoudaki M., Hartman A., Miladonovic H., Ngoc H.N., Karsten U., Gazera M. (2019). Bostrychines A-F, six novel micosporine-like amino acids and a novel betaine from the red alga *Bostrychia scorpioides*. Mar. Drugs.

[35] Huovinen P., Gómez I., Figueroa F.L., Ulloa N., Morales V., Lovengreen C. (2004). Ultraviolet-absorbing mycosporine-like amino acids in red macroalgae from Chile. Bot. Mar..

[36] Hoyer K., Karsten U., Wiencke C. (2002). Induction of sunscreen compounds in Antarctic macroalgae by different radiation conditions. Mar. Biol..

[37] Karsten U., Sawall T., Hanelt D., Bischof K., Figueroa F.L., Flores-Moya A., Wiencke C. (1998). An inventory of UV-absorbing mycosporine-like amino acids in macroalgae from polar to warm-temperate regions. Bot. Mar..

[38] Korbee-Peinado N. (2004). Fotorregulación y Efecto del Nitrógeno Inorgánico en la Acumulación de Aminoácidos Tipo Micosporina en Algas Rojas.

[39] Karsten U., Sawall T., West J., Wiencke C. (2000). Ultraviolet sunscreen compounds in epiphytic red algae from mangroves. Hydrobiologia.

[40] Briani B., Sissini M.N., Lucena L.A., Batista M.B., Costa I., Nunes J.M., Schmitz C., Ramlov F., Maraschini M., Korbee N.K.R. (2018). Mycosporine like amino acids (MAAs) in red marine algae and their relations with abiotic factors along southern Atlantic coast. J. Phycol..

[41] Schneider G., Figueroa F.L., Vega J., Chaves P., Álvarez-Gómez F., Korbee N., Bonomi-Barufi J. (2020). Photoprotection properties of marine photosynthetic organisms grown in high Ultraviolet exposure areas: Cosmeceutical applications. Algal Res..

[42] Sun Y., Zhang N., Zhou J., Dong S., Zhang X., Guoi L., Guop G. (2020). Distribution, contents, and types of mycosporine like amino acids (MAAs) in marine macroalgae and a data base for MAAs based on their characteristics. Mar. Drugs.

[43] Sinha R.P., Singh S.P., Häder D.-P. (2007). Database on mycosporines and mycosporine-like amino acids (MAAs) in fungi, cyanobacteria, macroalgae, phytoplankton and animals. J. Photochem. Photobiol. B.

[44] Hylander S. (2020). Mycosporine-like amino acids (MAAs) in zooplankton. Mar. Drugs.

[45] Jofre J., Celis-Plá P., Figueroa F.L., Navarro N.P. (2020). Seasonal variation of mycosporine-like amino acids in three subantarctic red seaweeds. Mar. Drugs.

[46] Vega J., Bonomi-Barufi J., Gómezz-Pinchetti J.L., Figueroa F.L. (2020). Cyanobacteria and Red Macroalgae as Potential Sources of Antioxidants and UV Radiation-Absorbing Compounds for Cosmeceutical Applications. Mar. Drugs.

[47] Rosic N.N. (2019). Mycosporine-like amino acids: Making the foundation for organic personalized sunscreens. Mar. Drugs.

[48] Chrapusta E., Kaminski A., Duchnik K., Bober B., Adamski M., Bialczyk J. (2017). Mycosporine-like amino acids: Potential health and beauty ingredients. Mar. Drugs.

